# Autologous minced cartilage repair for chondral and osteochondral lesions of the knee joint demonstrates good postoperative outcomes and low reoperation rates at minimum five-year follow-up

**DOI:** 10.1007/s00167-023-07546-1

**Published:** 2023-08-27

**Authors:** Armin Runer, Robert Ossendorff, Felix Öttl, Vincent A. Stadelmann, Stefan Schneider, Stefan Preiss, Gian M. Salzmann, Jakob Hax

**Affiliations:** 1 Department of Sports Orthopaedics, Klinikum rechts der Isar, Technical University of Munich, Ismaninger Str. 22, 81675 Munich, Germany; 2 Department for Orthopaedics and Trauma, University Hospital Bonn, Bonn, Germany; 3 Department of Research and Development, Schulthess Klinik, Zurich, Switzerland; 4 Orthocentrum Hamburg, Hamburg, Germany; 5 Department of Hip and Knee Surgery, Schulthess Klinik, Zurich, Switzerland

**Keywords:** Chondral lesion, Cartilage, Minced cartilage, Particulated cartilage, knee, Articular cartilage, Articular cartilage resurfacing, patient-reported outcomes

## Abstract

**Purpose:**

Minced cartilage is a one-step, autologous procedure with promising short-term results. The aim of the present study was to evaluate mid-term results in a patient cohort with chondral and osteochondral lesions in the knee joint treated with minced cartilage.

**Methods:**

From 2015 through 2016, a total of 34 consecutive patients were treated with a single-step, autologous minced cartilage for knee chondral and osteochondral lesions. Numeric analogue scale (NAS) for pain and knee function were obtained prior to surgery and at 12, 24 and 60 months postoperatively. Secondary outcomes, including Lysholm score, Tegner activity score, and the International Knee Documentation Committee (IKDC) score, were recorded at final follow-up. MRI examinations of patients with unplanned radiological follow-up were analysed using the MOCART (Magnetic Resonance Observation of Cartilage Repair Tissue) score.

**Results:**

A total of 28 patients (44.1% females, age at surgery: 29.5 ± 11.5 years) were available at a mean follow-up of 65.5 ± 4.1 months. Mean defect size was 3.5 ± 1.8 cm^2^. NAS for pain decreased from a median of 7 (range: 2–10) preoperatively to 2 (0–8) postoperatively. NAS knee function improved from a median of 7 (range: 2–10) to 3 (0–7) after five years, respectively. Satisfactory Lysholm (76.5 ± 12.5), IKDC (71.6 ± 14.8) and Tegner activity (4, range 3–9) scores were reported at final follow-up. Of all patients, 21(75%) and 19 (67.9%) reached or exceeded the PASS for the IKDC- and Lysholm score at final follow-up, respectively. The average overall MOCART 2.0 scores for all postoperatively performed MRIs (*n* = 23) was 62.3 ± 17.4. Four (14.2%) postoperative complications were directly linked to minced cartilage, one (3.5%) of which required revision surgery.

**Conclusion:**

One-step, autologous minced cartilage repair of chondral and osteochondral lesions of the knee without the necessity for subchondral bone treatment demonstrated good patient-reported outcomes, low complication rates, and graft longevity at mid-term follow-up. Minced cartilage represents a viable treatment option to more traditional cartilage repair techniques even in mid-term.

**Level of evidence:**

Level III.

## Introduction

The incidence of chondral and osteochondral lesions of the knee is increasing, but treatment remains challenging [13, 52]. Several different cartilage repair techniques have been described, each one aiming to maximize the amount of mature and organized hyaline or hyaline-like cartilage [7, 19]. However, so far, no technique has been able to regenerate normal hyaline cartilage in adults on a regularly basis and no technique has been proven to be superior. Osteochondral autograft transfer system (OATS), autologous chondrocyte implantation (ACI), matrix-assisted chondrocyte implantation (MACI) or several cartilage repair techniques using scaffolds (natural or synthetic) are contemporarily the most commonly therapies for medium to large defects [7, 22]. ACI and MACI are purported to generate hyaline or hyaline-like cartilage, with low associated reoperation rates and favourable clinical outcomes even in complex cases [5, 7, 18, 29, 49]. However, the two-step approach of ACI and MACI, which requires laboratory cell cultivation, results in a high financial and clinical burden. OATS results are reported to be similar to MACI, but allografts availability may be limited and cost intensive.

Recently, the minced cartilage procedure, a technique in which viable autologous cartilage is collected, sliced into small pieces, and reimplanted, has undergone renewed interest [51, 55]. Autologous thrombin and PRP, fibrin glue or a membrane might be additionally used for fragment fixation [42, 50–52]. Short-term outcomes in literature have been promising, but mid- and long-term results are lacking but urgently needed [8, 10, 42].

The aim of the present single-cohort study is to demonstrate minimum five-year outcome data of a cohort that underwent minced cartilage for the treatment of chondral and osteochondral lesions in the knee. It has been hypothesized that minced cartilage may maintain favourable clinical results over longer term follow-up, both in terms of patient-reported outcome measures and reoperation rates.

## Materials and methods

Local ethical committee of the Canton of Zurich (KEK-ZH-Nr. 2015–0258) approval was obtained prior to study initiation. Informed consent was signed by all participants. The first 34 consecutive patients who underwent minced cartilage knee surgery between 2015 and 2016 were retrospectively analysed from a prospectively maintained database that routinely collected patient-related outcome data.

All patients were contacted personally by telephone for an interview and questionnaires were collected by mail. Electronic medical records were reviewed to obtain patient demographic, surgical, and imaging data.

### Surgical procedure

Indication and detailed surgical treatment regimen was described previously [42]. All surgical interventions were performed by a single specialized and fellowship-trained orthopaedic surgeon (G.M.S.). All patients obtained a preoperative Magnetic Resonance Imaging (MRI) and conventional knee joint radiographs for diagnostic and surgical planning purposes. Patients with chondral or osteochondral lesions of the knee, who did not require any subchondral bone treatment were included in this study. Additionally, those with osteochondritis dissecans lesions not amenable to primary fixation, with otherwise healthy-appearing cartilage in the remaining compartments were included.

Final indication for performing a minced cartilage procedure was made following routine arthroscopy. In all cases, the second-generation repair technique was used as described previously [42, 50]. In brief, depending on the location of the chondral defect, a medial or lateral mini arthrotomy approach was performed and the lesion was inspected and measured. The defect was then debrided with a curette until a stable healthy cartilage rim was obtained. The healthy hyaline cartilage obtained from the debridement was collected and subsequently minced into small fragments of approximately 1 mm until a paste-like consistency was achieved. If an insufficient amount of cartilage was obtained, additional healthy cartilage was harvested from intercondylar notch using osteochondral cylindrical harvesters. Finally, minced cartilage was placed into the defect and sealed with fibrin glue or a combination of fibrin glue and membrane (Chondro-Gide, Geistlich Pharma).

### Rehabilitation

An identical postoperative rehabilitation protocol was used in all patients with initial bed rest in a straight knee brace for 24 h. Continuous passive motion machine was started on the first postoperative day. For the first six weeks, partial weight-bearing was permitted with crutches and range of motion was limited to 0 to 90 degrees. After six weeks, a gradual increase in weight-bearing and range of motion was permitted, with full weight-bearing and unrestricted range of motion achieved at approximately nine weeks postoperatively.

### Patient-reported outcome measures

Primary patient-reported outcome measures (PROMs), obtained preoperatively and at 12, 24 and 60 months of follow-up, were the numeric analogue scale (NAS) for pain and subjective knee function (0 = no pain/best function, 10 = worst pain/worst function). Secondary PROMs, including Lysholm, IKDC (International Knee Documentation Committee), COMI (Core Outcome Measurement Index) and Tegner activity score, were obtained at final follow-up only, as they were not routinely captured preoperatively from the patient-reported outcome data system. All postoperative complications and reoperations were recorded.

### Radiological outcome measures

Preoperative 3-T MRI scans obtained at our institution were evaluated by a trained and blinded examiner using the AMADEUS (Area Measurement and Depth and Underlying Structures) score in order to quantify the severity of chondral and osteochondral defects prior to cartilage repair [33]. Intraoperative grading was performed according to the ICRS (International Cartilage Repair Society) grading system [3]. Hyaline cartilage or repair tissue were analysed using the MOCART (magnetic resonance observation of cartilage repair tissue, 0 = worst, 100 = best) score in all patients at 6 month postoperatively [40]. Preoperative and six-month postoperative outcomes have been published previously and are therefore not reported in the present study [42]. In addition to the planned study MRIs, all subsequent, unscheduled MRI examinations were captured as part of this study and analysed using MOCART 2.0 score by a single investigator who was blinded to the clinical outcome of the patients. The MOCART 2.0 score has been shown to have excellent interrater and intrarater reliability [53].

### Statistical analysis

Statistical analysis was performed using SPSS (Version 28, IBM) and Microsoft Excel (Version 16). Normal distribution of the data was tested using the Kolmogorov–Smirnov test. Friedman test for dependent samples with Dunn-Bonferroni post-hoc test was used to compare NAS values at different study time points. Student’s *t* test or Mann–Whitney *U* test was applied to determine differences between groups. The patient-acceptable symptomatic state (PASS) threshold was employed as a tool to assess the minimum scores associated with patient satisfaction [30]. In cartilage repair, a final IKDC score of 62.1 and a Lysholm score of 70 have been reported to correspond with the PASS [6]. A difference between IKDC- and Lysholm score values greater than 9.2 and 13.0, respectively, was considered a clinically important difference (CID) [6]. Using G*Power (Version 3.1), the Wilcoxon signed-rank test for matched pairs with maximum correlation between the pre- and postoperative groups was used for a-priori sample size calculation. With *α*-level set to 0.05, *β* to 0.80, and assuming a one-tailed analysis, a total of 28 patients were deemed necessary to detect a one-point difference with two points standard deviation in the NAS for pain score. One-point difference in NAS for pain is within the reported minimal clinically important difference (MCID) of 2.7 points for cartilage procedures [32]. With an expected loss to follow-up rate of 20% at mid- to long-term, the first 34 consecutive patients were included in the study.

## Results

A total of 34 consecutive patients treated with minced cartilage were included in the study. A detailed overview of patient characteristics and concomitant procedures is shown in Table 1. Cartilage defect characteristics are displayed in Table 2. The final follow-up after a mean of 65.5 ± 4.1 months was 82.4% (*n* = 28). Patients lost to follow-up did not differ in baseline characteristics or in intraoperative findings to those with complete mid-term follow-up.

**Table 1:** Patient characteristics and intraoperative details

Number patients (% female)	34 [44.1]
Age	29.5 ± 11.4
Height (cm)	174.3 ± 10.0
Weight (kg)	74.7 ± 16.0
BMI	24.4 ± 3.5
Active Smokers at time of surgery	8 (23.5)
Preoperative MRI	
Preoperative AMADEUS score	55.2 ± 21.5
Chondral lesions	21 (61.8)
Osteochondral lesions	13 (38.2)
Concomitant procedures^a^	
None	17 (50)
MPFL	4 (11.8)
Insall procedure	3 (8.8)
High tibial/distal femoral osteotomy	2 (5.9)
ACLR	1 (2.9)
Epiphysiodesis	1 (2.9)
ORIF	1 (2.9)
Plate/screw removal	1 (2.9)
Lateral lengthening	1 (2.9)
Intraoperative MC fixation	
Fibrin glue	14 (41.2)
Membrane and Fibrin glue	20 (58.8)

Data displayed as number (per cent) or mean ± standard deviation

*MRI* magnet resonance imaging, *FC* femoral Condyle, *BMI* body mass index, *MPFL* Medial patellofemoral ligament, *ORIF* open reduction and internal fixation, *ACL* anterior cruciate ligament reconstruction

^a^Multiple procedures possible; AMADEUS, Area Measurement and Depth and Underlying Structures

**Table 2:** Defect characteristics based on MRI and intraoperative findings

Defect location^a^	n (%)
Retropatellar	19 (55.9)
Medial FC	5 (14.7)
Lateral FC	5 (14.7)
Medial + lateral FC	1 (2.9)
Medial FC + retropatellar	1 (2.9)
Lateral FC + retropatellar	1 (2.9)
Trochlear groove	1 (2.9)
Tibial	1 (2.9)
Appearance on MRI	
Defect size	
≤ 1cm^2^	4 (12.9)
> 1cm^2^ ≤ 2cm^2^	17 (54.8)
> 2cm^2^ ≤ 4cm^2^	6 (19.4)
> 4cm^2^ ≤ 6cm^2^	4 (12.9)
≥ 6cm^2^	0 (0)
Chondral defect	
(a) Signal alteration	2 (6.5)
(b) Partial-thickness defect	17 (54.8)
(c) Full-thickness defect	12 (38.7)
Intraoperative appearance	
Defect size	
≤ 1cm^2^	3 (8.8)
> 1cm^2^ ≤ 2cm^2^	7 (20.4)
> 2cm^2^ ≤ 4cm^2^	12 (35.2)
> 4cm^2^ ≤ 6cm^2^	8 (23.5)
≥ 6cm^2^	4 (11.7)
ICRS grading	
Grade 1	0 (0)
Grade 2	0 (0)
Grade 3	19 (61.3)
Grade 4	12 (38.7)

Data displayed as number (per cent)

*FC* femoral Condyle, *ICRS* International Cartilage Repair Society, *MRI* magnetic resonance imaging

^a^Multiple locations possible

### Patient-reported outcome measurements

**Fig. 1:**
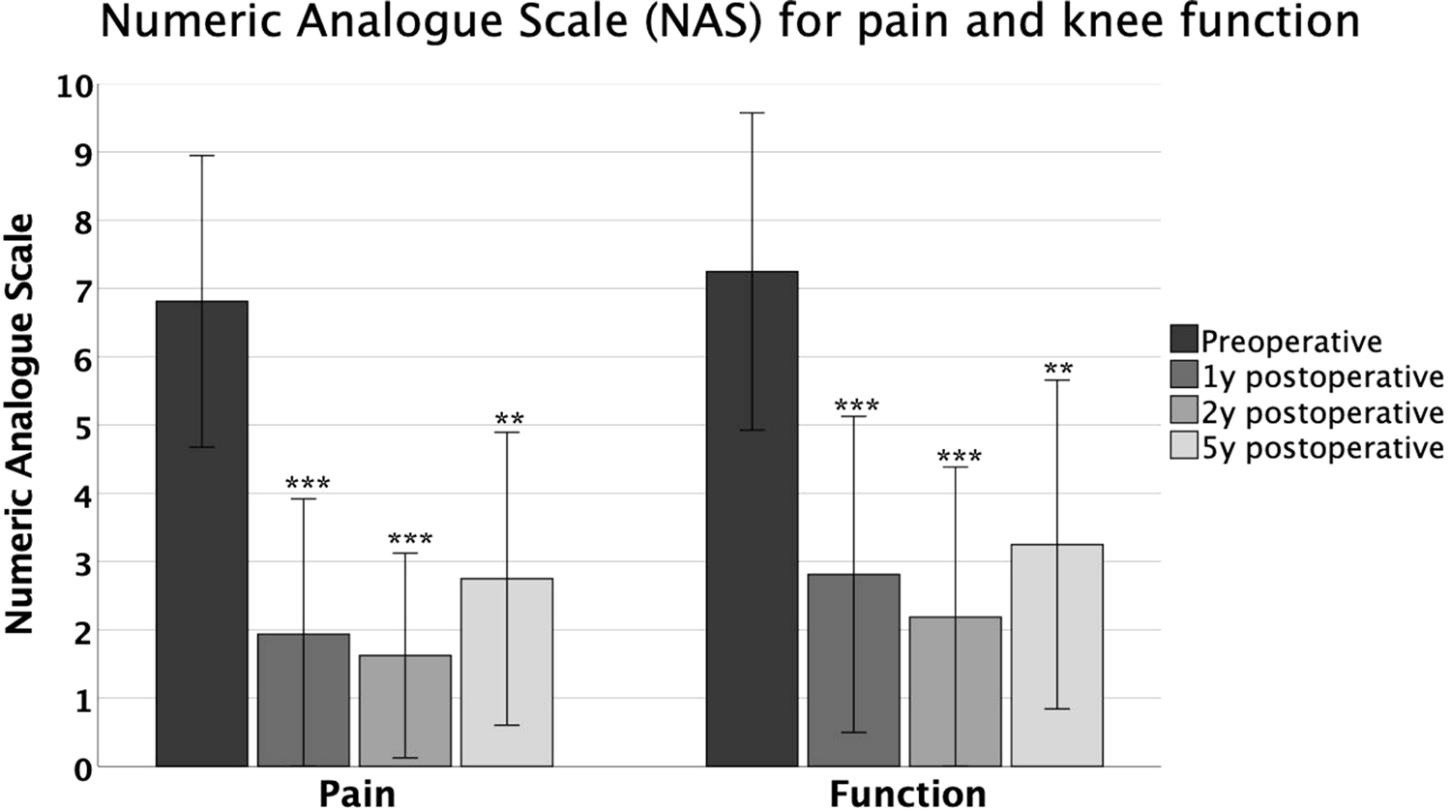
Numeric analogue scale (NAS) for pain and knee function preoperative and one, two and five years postoperative. The asterisk marks a significant difference to the preoperative state: ***p < 0.001; **p < 0.01. No significant differences were observed between 1, 2 and 5 year postoperative follow-up. MCID: NAS for pain: 2.7

Overall, a statistically significant decrease in NAS for pain with a medium to strong effect size and a statistically significant (*p* < 0.001) increase in knee function with a medium to strong effect size were observed throughout the study period (Fig. 1). There was no statistically significant increase in postoperative pain or worsening of knee function at any of the three postoperative follow-up time points (Fig. 1).

Gender, defect localization, concomitant interventions, defect characteristics as well as fixation techniques for minced cartilage seem to have little influence on postoperative outcomes with similar PROMs reported (Table 3). Statistical subgroup analysis was not performed because of missing power.

**Table 3:** Five-year outcomes after minced cartilage procedure for different subgroups

	Total (*n* = 28)	Gender		Defect localization			Isolated vs combined procedures		Defect characteristic		Minced cartilage fixation	
		Male (*n* = 15)	Female (*n* = 13)	PF comp (*n* = 19)	TF comp (*n* = 8)	Tibial (*n* = 1)	Isolated (*n* = 14)	Combined (*n* = 14)	Chondral (*n* = 17)	Osteo-chondral (*n* = 11)	Fibrin Glue (*n* = 15)	Membrane + Fibrin Glue (*n* = 13)
NAS for pain*	2 (0–8)	2 (0–5)	3 (0–8	2 (0–5)	2 (0–8)	3 (0–3)	2 (0–5)	2.5 (0–8)	2 (0–5)	2 (0–8)	2 (0–8)	3 (1–5)
NAS for function*	3 (0–7)	3 (0–7)	4 (0–7)	3 (0–7)	4 (0–7)	3 (1–4)	4 (1–7)	2.5 (0–7)	3 (0–7)	3 (0–7)	4 (0–7)	3 (1–7)
Lysholm score (CID = 13)	76.5 ± 12.5	80.1 ± 9.8	73.9 ± 13.8	76.6 ± 13.8	76.6 ± 8.4	88.0	79.1 ± 12.5	78.1 ± 13.3	79.2 ± 11.5	72.0 ± 13.3	78.8 ± 13.7	73.5 ± 10.4
IKDC score (CID = 9.2)	71.6 ± 14.8	75.7 ± 9.7	68.3 ± 17.7	73.1 ± 17.0	70.3 ± 8.8	67.8	75.4 ± 16.5	74.2 ± 13.3	73.4 ± 13.0	68.1 ± 18.3	71.8 ± 17.4	71.5 ± 11.8
COMI score	2.2 ± 1.9	1.8 ± 1.7	2.6 ± 2.2	2.0 ± 2.1	2.7 ± 1.6	3.4	1.9 ± 1.6	1.6 ± 1.4	1.8 ± 1.3	3.1 ± 2.7	2.1 ± 2.2	2.3 ± 1.5
Tegner activity score*	4 (3–9)	4 (3–9)	4 (3–7)	6 (3–9)	3.5 (3–4)	4 (3–9)	4 (3–7)	4 (3–9)	4 (3–7)	4 (3–9)	4 (3–9)	4 (3–9)

Data displayed as mean ± standard deviation if not otherwise stated

* NAS* numeric analogue scale, *IKDC* International Knee Documentation Committee, *COMI* Core Outcome Measures Index, *PF comp* Patellofemoral Compartment, *TF comp* Tibiofemoral Compartment, *CID* Clinically Important Difference

^a^Displayed as median (minimum–maximum)

Of all patients 75% (*n* = 21) and 68% (*n* = 19) reached or exceeded the PASS for the IKDC- and Lysholm score at final follow-up, respectively.

### Surgery-related complications and revision surgery

A total of four (14.2%) surgery-related complications were related directly to minced cartilage, one (3.5%) of which required revision surgery whereas the others (10.7%) resolved without surgical intervention. Five (17.8%) adverse events were related to the additional performed procedures (e.g. ACL reconstruction, MPFL reconstruction, etc.) and one (3.5%) event was linked to a traumatic accident (Table 4). If the assumption is made that all patients lost to follow-up had complications, the total complication rate would be 47.0%.

**Table 4:** Surgery-related complications and subsequent interventions

AE #	Initial Surgery	Revision surgery required	Type of Complication	Subsequent Intervention	Time after surgery (month)	AE Grade
Complications directly related to minced cartilage procedure						
1	Minced cartilage procedure and lateral meniscectomy	No	Persistent pain	Single intraarticular corticosteroid infiltration	19	2
2	Minced cartilage procedure and medial meniscectomy	No	Slight pain and mechanical symptoms due to hypertrophic cartilage	None	60	1
3	Minced cartilage procedure including spongiosaplastic	Yes	Persistent cartilage damage, plica infrapatellaris	Plica removal and revision minced cartilage procedure	60	3
4	Minced cartilage procedure	No	Delayed wound healing	None	1	1
Complication not related to minced cartilage procedure						
5	MPFL reconstruction and minced cartilage procedure	Yes	Medial tightness and pain, persistent retropatellar crepitus	MPFL release	16	3
6	MPFL reconstruction and minced cartilage procedure	Yes	Partial MPFL graft failure and retropatellar scarring	Arthroscopy and debridement	6	3
7	Minced cartilage procedure and ACLR	No	Motion deficit	Physical therapy	5	2
8	Minced cartilage procedure and ORIF proximal tibia	Yes	Motion deficit, osteophytosis	Arthroscopy, arthrolysis, osteophyte removal	3	3
9	Minced cartilage and distal femoral osteotomy	No	Delayed wound healing at osteotomy site	None	1	1
10	Minced cartilage procedure	Yes	Trauma accident with chondral injury	Surgical treatment with minced cartilage procedure	44	

*AE* adverse event, *#* number of complications, *MC* minced cartilage, *MPFL* medial patellofemoral ligament, *ACLR* anterior cruciate ligament reconstruction, *ORIF* Open reduction internal fixation

### Radiological outcomes

Nineteen (67.8%) patients obtained a total of 23 unscheduled MRI at a mean of 41.8 ± 22.0 month after surgical intervention. Reasons for MRI included trauma, revision surgery, or pain (Table 5). The average overall MOCART 2.0 score including all examined patients and all anatomical sites (retropatellar, femoral condyle, trochlea) was 62.3 ± 17.4. MOCART 2.0 results including detailed information about all variables for each anatomical site and follow-up time point are shown in Table 5.

**Table 5:** MOCART 2.0 score of unscheduled MRI at 2–5 year of follow-up

	within 2 years FU	within 3 years FU	within 4 years FU	within 5 years FU	Overall
Number of patients^$^	12	2	4	7	23
Location (retropatellar/FC/Trochlea)^a^	7/5/1	1/0/1	2/1/1	4/5/0	14/11/3
Chondral lesion ICRS-Grade I/II/III/IV	0/1/8/0	0/0/1/0	0/0/3/0	0/0/3/0	0/1/15/0
Osteochondral lesion (Burns) Grade I/II/III/IV	0/0/2/2	0/0/0/1	0/0/0/1	0/0/3/3	0/0/5/7
Cause for follow up MRI (Trauma/Symptoms/Routine/NA)	0/3/6/4	0/2/0/0	0/3/0/1	4/3/1/1	4/11/7/6
MOCART 2.0 Score					
Average MOCART 2.0 score	64.2 ± 15.8 (45–95)	37.5 ± 2.5(35–40)	50.0 ± 7.9 (40–60)	70.6 ± 16.7 (40–90)	62.3 ± 17.4
Retropatellar	65.7 ± 13.5 (45–85)	40 ± 0 (40)	57.5 ± 2.5 (55–60)	81.25 ± 4.1 (75–85)	67.1 ± 14.8
Femural condyle	61 ± 19.3 (45–95)		45 ± 0 (45)	62.0 ± 18.0 (40–90)	60.0 ± 18.5
Trochlea	70.0 ± 0 (70)	35 ± 0 (35)	40 ± 0 (40)		48.3 ± 15.5
Detailed Variables of MOCART 2.0					
Volume of defect repair and defect filling	18.0 ± 3.7 (10–20)	15 ± 5 (10–20)	15.0 ± 6.1 (5–20)	17.2 ± 4.1 (10–20)	17.1 ± 4.5
Subclasses (1/2a/2b/3/4/5a/5b)	10/1/0/2/0/0/0	1/0/0/1/0/0/0	2/1/0/0/1/0/0	6/1/0/2/0/0/0	20/3/0/5/0/0/0
Integration to adjacent cartilage	10.4 ± 2.4 (5–15)	7.5 ± 2.5 (5–10)	7.5 ± 2.5 (5–10)	11.7 ± 3.3 (5–15)	10.2 ± 3.1
Surface of the repair tissue	5.4 ± 1.3 (5–10)	5.0 ± 0 (5)	3.75 ± 2.2 (0–5)	7.2 ± 2.5 (5–10)	5.7 ± 2.2
Structure of the repair tissue	1.5 ± 3.6 (0–10)	0 ± 0 (0)	1.3 ± 2.2 (0–5)	0.6 ± 1.6 (0–5)	1.1 ± 2.8
Signal intensity of the repair tissue	10.4 ± 3.6 (0–15)	10.0 ± 0 (10)	7.5 ± 4.3 (0–10)	11.7 ± 2.4 (10–15)	10.3 ± 3.5
Subclasses (1/2a/2b/3a/3b)	3/6/3/0/1	0/2/0/0/0	0/3/0/0/1	3/2/4/0/0	6/13/7/0/2
Bony defect or bony overgrowth	6.2 ± 2.9 (0–10)	0 ± 0 (0)	3.7 ± 4.1 (0–10)	7.8 ± 2.5 (5–10)	5.9 ± 3.5
Subclasses (1/2a/2b/3a/3b)	4/6/2/1/0	0/0/0/2/0	1/1/0/1/1	5/4/0/0/0	10/11/2/4/1
Subchondral changes	12.3 ± 7.5 (55–85)	0 ± 0 (0)	11.2 ± 7.3 (0–20)	14.4 ± 6.4 (0–20)	12 ± 7.7
Subclasses (1/2/3/4a/4b)	4/4/2/3/0	0/0/0/2/0	1/1/1/1/0	4/2/2/1/0	9/7/5/7/0

All values displayed as mean ± standard deviation (range) if not otherwise stated

*FC* femoral condyle, *MOCART* Magnetic Resonance Observation of Cartilage Repair Tissue, *$* Four patients obtained two, and one patients three MRIs

^a^Multiple location per patient possible

## Discussion

The primary finding of the study was that second-generation minced cartilage procedure for the treatment of chondral and osteochondral lesions of the knee without the necessity for subchondral bone treatment is an effective and safe procedure with good mid-term results in terms of pain and knee function. The present 5-year data demonstrated no significant worsening of pain and function at mid-term compared to short-term follow-up.

The primary aim of minced cartilage is to recapitulate hyaline or hyaline-like cartilage at site of implantation from cell outgrowth, proliferation, and differentiation without the need for a two-step surgical process. Fragmentation of healthy cartilage has been shown to “activate” chondrocytes by increasing tissue surface and therefore promoting outgrowth [37, 51]. Outgrowth of chondrocytes results in proliferation and matrix production. This process is believed to be positively influenced by native joint physical-biomechanical inputs and the osteochondral microenvironment, as mechanical and biological stimuli have been shown to promote proliferation and chondrogenic differentiation [51, 56]. Several animal models have demonstrated the feasibility of minced cartilage showing better results compared to microfracturing, while demonstrating similar outcomes to two-stage autologous chondrocyte implantation [1, 9, 15, 26, 38, 41].

As it currently stands, there is only one prospective randomized clinical trial for single-stage autologous cartilage fragments procedure (CAIS, Cartilage Autograft Implantation System) [10] and three trials involving use of autologous minced cartilage with clinical follow-up of up to 24 months. [8, 12, 42]. Comparing two-year outcomes of patients randomly treated with either microfracturing (MFX) or CAIS shows significantly higher PROMs for CAIS [10]. There was no difference in the number of surgery-related complications, but a higher number of intralesional osteophyte formation in patients treated with MFX [10]. A statistical significant increase in MOCART scores and PROMs were also reported in eight patients with osteochondrosis dissecans treated with a combination of autologous bone and cartilage chips (ADTT, autologous dual-tissue transplantation) [8]. Similar, a statistically significant improvement between pre- and postoperative PROMs values were reported in fifteen patients treated with a novel autologous-made matrix, hyaline cartilage chips and platelet-rich growth factors [12]. In the present study, five-year outcomes indicate maintained low pain scores and knee function which are not statistically different from one- and two-year postoperative outcomes. Despite a slight increase in pain compared to one- and two-year results, the NAS pain values were within the limits of the minimal clinically important difference (MCID) and not statistically significantly different. Further long-term data are needed to assess the postoperative progression over time.

When comparing outcomes of different studies it is important to keep in mind, that several factors including surgical techniques, patient characteristics [48], previous or subsequent surgical interventions [48], defect size and location [23] as well as rehabilitation [20] have an influence on the postoperative outcome and limit therefore the direct comparability. Recently two-stage autologous chondrocyte implantation or osteochondral allograft transplantation (OATS), research showed promising mid-term results after MACI for medium to large defects [20, 24, 31]. A recent prospective randomized controlled trial (RCT) comparing MFX (61.8 ± 21.5) to MACI (68.5 ± 21.2) reported significant higher IKDC scores for the latter [4]. These findings are supported by recent meta-analysis showing no increased risk of clinical failure but superior improvements in PROMs for MACT compared to MFx at short- to mid-term [14] [20]. Contrary, other studies question the superiority of MACI over MFX [34, 35] or even report superiority of MFX especially in patients with only small chondral defects [47]. A prospective, controlled clinical trial studying the safety and efficacy of MACI with spheroid technology reported good and stable improvement of IKDC (74.6 ± 18.7) and KOOS (77.1 ± 18.6) scores after 48 months [46]. This is in accordance with several other mid- to long-term studies reporting stable improvements after ACI [2, 4, 11, 24, 36, 45, 57] When directly comparing ACI to OATS [43] fair to good mid-term IKDC outcomes (IKDC: 50 – 80) were reported [21]. Similarly, no significant differences at mid-term were found in PROM regarding outcomes between ACI and AMIC with VAS for pain scores ranging between 2.3 – 3- two years postoperative [25, 27, 54]. Patient-reported outcomes of the present study (IKDC: 71.6 ± 17.8) are comparable to MACI, OATS and AMIC results of the above-mentioned studies but appear to be higher than those reported for MFX [4]. Gender, concomitant injuries, defect localization, defect characteristics as well as fixation techniques for minced cartilage seem to have little influence on postoperative outcomes. Patients treated with one-staged minced cartilage for chondral and osteochondral knee defects can expect similar postoperative outcomes in the mid-term as compared to other established procedures.

Overall surgery-related complications and revision surgery are relatively low and comparable between different chondral regenerative techniques [18, 28, 44, 46]. Graft hypertrophy after MACI was reported in 12% of the patients after five years with a need for revision surgery in 8% [16]. Similar revision surgery rates were reported after five years in a prospective RCT rates comparing MACI (10.8%) and MFX (9.5%) [4]. In contrast, no graft hypertrophy was observed in a study using ACI (Spherox™, CO.DON AG, Germany; formerly known as chondrosphere) [45]. Mid-term outcome analysis of OATS revealed an 87% graft survival rate at 5 years with a 37% reoperation rate. These results were supported by a recent systematic review reporting similar survival rates (87%) and reoperation rates (30%) at mid- to long-term. In the present study, a total of four (14.2%) complications were primarily related to the minced cartilage procedure, one of which required revision surgery whereas the others resolved without surgical intervention. No revision operation was necessary due to graft hypertrophy or mechanical symptoms related to the chondral graft, although one patient reported still of occasional joint locking at 5 years, possibly related to graft hypertrophy. Altogether, this makes minced cartilage comparable to MFX and ACI in terms of surgery-related complication and revision surgery, and overall a safe and efficient procedure.

Despite the well-known lack of correlation between postoperative radiological outcomes and PROMs, radiological scoring remains relevant as a measure of structural change over time [17]. Two prospective randomized controlled trials reported mean MOCART scores of 76 ± 16 at two years and 75.5 ± 13.1 after 4 years, respectively [45, 46]. When comparing patellar and femoral condyle defects similar MOCART scores were observed [39, 58]. In the present study, only unscheduled follow-up MRIs within two and five years postoperatively were included, that were obtained for re-presentation due to recent trauma, pain, or revision surgery. Naturally, slightly lower MOCART 2.0 scores were therefore observed compared to the above-mentioned studies. Due to the nature of follow-up MRI indications in the present study, it can be assumed that MOCART 2.0 scores would be higher if the whole study cohort was included.

This work has some limitations. First, this study presents mid-term outcomes of the first series of patients undergoing minced cartilage of a single surgeon and therefore lacks a comparative group of patients treated with alternative osteochondral procedures such as ACI, OATS or MTX. Second, as consecutive patients were included and inclusion criteria were not limited to isolated cartilage defects, the heterogeneity of the study population might pose a limitation. However, a recent systematic review demonstrated good clinical outcomes after cartilage repair at the patellofemoral joint even in complex cases [5]. Third, the majority of the cartilage defects were located at the patella or femoral condyle and only a few at the trochlea or tibia. Therefore, the present results may not be applied without restriction to all cartilage defect locations in the knee joint. Moreover, overall outcomes might be lower, as less satisfactory results are for patients affected by cartilage lesions of the patella compared to other sides [23]. Due to the small sample size, statistical subgroup analyses were not possible, but the descriptive statistics appear to be comparable between the different defect localizations. Finally, while all patients obtained a preoperative and a six-month postoperative MRI scan, an additional, mid-term radiological examination for all patients would have been desirable; however, this was beyond the scope of the present work.

Minced cartilage procedure for the treatment of medium to large chondral and osteochondral defects of the knee, show good and promising results at mid-term. Minced cartilage procedure seems to be a viable treatment alternative in patients where a two-staged approach is not desired.

## Conclusion

Patients treated with autologous minced cartilage procedure for medium to large chondral and osteochondral lesions of the knee without the necessity for subchondral bone treatment report good mid-term results in pain and knee function and low rates of postoperative adverse events. Pain and functional levels remain stable and within the MCID at mid-term.

## Data Availability

Not applicable.
